# Reduced Lipopolysaccharide-Binding Protein (LBP) Levels Are Associated with Non-Alcoholic Fatty Liver Disease (NAFLD) and Adipose Inflammation in Human Obesity

**DOI:** 10.3390/ijms242417174

**Published:** 2023-12-06

**Authors:** Ilaria Barchetta, Flavia Agata Cimini, Federica Sentinelli, Caterina Chiappetta, Claudio Di Cristofano, Gianfranco Silecchia, Frida Leonetti, Marco Giorgio Baroni, Maria Gisella Cavallo

**Affiliations:** 1 Department of Experimental Medicine, Sapienza University of Rome, 00185 Rome, Italy; ilaria.barchetta@uniroma1.it (I.B.); flaviaagata.cimini@uniroma1.it (F.A.C.); 2 Department of Public Health and Infectious Diseases, Sapienza University of Rome, 00185 Rome, Italy; federica.sentinelli@uniroma1.it; 3 Department of Medical-Surgical Sciences and Bio-Technologies, Sapienza University of Rome, 04100 Latina, Italy; caterina.chiappetta@uniroma1.it (C.C.); claudio.dicristofano@uniroma1.it (C.D.C.); frida.leonetti@uniroma1.it (F.L.); 4 Department of Medical and Surgical Sciences and Translational Medicine, Faculty of Medicine and Psychology, St Andrea Hospital, Sapienza University of Rome, 00189 Rome, Italy; gianfranco.silecchia@uniroma1.it; 5 Endocrinology and Diabetes, Department of Clinical Medicine, Public Health, Life and Environmental Sciences (MeSVA), University of L’Aquila, 67100 L’Aquila, Italy; marcogiorgio.baroni@univaq.it; 6 Neuroendocrinology and Metabolic Diseases, IRCCS Neuromed, 86077 Pozzilli, Italy

**Keywords:** low-grade inflammation, insulin resistance, type 2 diabetes, NASH, MAFLD, MASLD, liver fibrosis, visceral obesity, metabolic syndrome

## Abstract

Lipopolysaccharide (LPS) and its binding protein LBP have emerged as potential contributors to the progression from overweight/obesity to overt metabolic diseases and NAFLD. While LPS is known to activate hepatocyte inflammation, thus contributing toward NAFLD development, the role of LBP is more intricate, and recent data have shown that experimental reduction in hepatic LBP promotes NAFLD progression. In this cross-sectional investigation, we evaluated circulating LBP in relation to obesity, NAFLD, visceral adipose tissue (VAT) inflammation, and type 2 diabetes (T2D). We recruited 186 individuals (M/F: 81/105; age: 47 ± 10.4 years; BMI: 35.5 ± 8.6 kg/m^2^); a subgroup (*n* = 81) underwent bariatric surgery with intra-operative VAT and liver biopsies. LBP levels were higher in obese individuals than non-obese individuals but were inversely correlated with the parameters of glucose metabolism. Reduced LBP predicted T2D independent of age, sex, and BMI (*p* < 0.001). LBP levels decreased across more severe stages of hepatosteatosis and lobular inflammation, and were inversely associated with VAT inflammation signatures. In conclusion, LBP levels are increased in obese individuals and are associated with a more favorable metabolic profile and lower NAFLD/NASH prevalence. A possible explanation for these findings is that hepatic LBP production may be triggered by chronic caloric excess and facilitate LPS degradation in the liver, thus protecting these individuals from the metabolic consequences of obesity.

## 1. Introduction

The prevalences of metabolic diseases, including obesity, non-alcoholic fatty liver disease (NAFLD), and type 2 diabetes mellitus (T2DM), have reached epidemic proportions worldwide, posing significant health challenges [1]. A dysmetabolic status is associated with chronic low-grade inflammation, which also triggers and worsens the severity of insulin resistance, leading to overt metabolic and cardiovascular diseases [2].

Despite the well-described clinical connection between systemic inflammation and metabolic dysfunction, the interplay between metabolic dysregulation and inflammatory processes is extremely intricate and has gained considerable attention in the quest to unravel the underlying mechanisms contributing to disease development and progression.

These aspects are of remarkable importance when investigating the pathophysiology and clinical outcomes of NAFLD, a hepatic hallmark of the metabolic syndrome, which is detectable in over 32% of the general adult population and associated with the whole spectrum of metabolic disturbances [1,2]. Regarding the tight connection between NAFLD and the presence of insulin resistance, recent consensus reports have proposed and adopted novel NAFLD nomenclatures, such as metabolic dysfunction-associated fatty liver disease (MAFLD) [3] and, very recently, MASLD (metabolic dysfunction-associated steatotic liver disease), the latter highlighting the need for only steatosis plus any single cardiometabolic criterion—among visceral obesity, impaired glucose metabolism, blood hypertension, and atherogenic dyslipidemia—to make the diagnosis of metabolic hepatopathy [4].

NAFLD is prevalent in 70–80% of T2DM individuals, exacerbating diabetes complications [5]. However, its pathogenesis remains unclear, and validated therapies are lacking, aside from lifestyle interventions [6]. The “multiple-hit hypothesis” suggests that NAFLD results from various factors acting on a backdrop of chronic caloric excess and insulin resistance, leading to intra-hepatocyte lipid accumulation, inflammation, and liver fibrosis [7]. Recent evidence highlights the crucial role of gut–liver axis disruption in metabolic imbalance, primarily driven by chronic low-grade inflammation, lipotoxicity, and insulin resistance [8]. In this context, lipopolysaccharide (LPS) and LPS-binding protein (LBP) have emerged as potential mediators in the progression to metabolic disorders and NAFLD [9,10].

LPS, found in Gram-negative bacterial outer membranes, consists of lipids and polysaccharides. Dietary and microbiota imbalances lead to intestinal Gram-negative bacterial overgrowth, resulting in increased LPS accumulation and serum endotoxemia. LPS enters the bloodstream via the intestinal mucosa, triggering inflammatory responses via the Toll-like receptor 4 (TLR4). Upon reaching the liver, LPS activates TLR4-mediated inflammation in hepatocytes, contributing to NAFLD development in overweight/obese individuals [9], and it also acts through epigenetic pathways by inhibiting DNMT3B expression and enhancing triglyceride synthesis and lipid droplet formation [10].

LBP is a 58 kDa glycoprotein produced by the liver and acts as a type I acute-phase molecule; its serum levels peak shortly after bacteremia onset and remain elevated for up to 72 h [11,12,13]. Once in the bloodstream, LBP forms a complex with LPS and promotes LPS binding to CD14 receptors, which in turn are linked to TLR4s, initiating the inflammatory cytokine cascade [14]. Thus, LBP takes part in the complex mechanisms that regulate immune responses and involve the up- and down-regulation of inflammatory processes triggered by LPS.

Unlike the well-established effects of LPS, the influence of LBP on metabolic disorders and NAFLD is more complex. While some studies associated higher LBP levels with obesity [15,16,17], other recent investigations in obese youths [18] and adults [19] found no relation between LBP and metabolic abnormalities. In longitudinal investigations, LBP was associated with parameters of body adiposity in children but was neither correlated with metabolic alterations nor predicted the development of dysmetabolic disease later in life [20]. Recent findings even suggest that higher LBP levels might reduce cardiovascular risk in older adults [21].

Furthermore, experimental models support LBP’s potential protective role in metabolic diseases [22]. Experimentally reduced liver LBP levels, either under standard or non-obesogenic conditions, exacerbate liver inflammation, fibrosis, and oxidative stress in non-alcoholic steatohepatitis (NASH) in mouse models and are correlated with human liver damage markers, indicating a potential role for liver LBP in promoting liver health and mitigating NASH progression [22].

Indeed, despite extensive research, the connection between circulating LBP levels and metabolic diseases, beyond their link to increased body adiposity, remains inconclusive. Thus, in this study, we aimed to evaluate LBP levels in relation to the presence of obesity and to investigate the association between circulating LBP and key metabolic disease parameters, including NAFLD, adipose tissue inflammation, and the presence of T2DM.

## 2. Results

### 2.1. Serum LBP Predicts Greater Adiposity but Not Overt Metabolic Diseases and T2DM

In our study sample (*n* = 186), LBP levels were higher in obese individuals than non-obese individuals (33.4 ± 23.2 vs. 16.6 ± 10.7 μg/mL, respectively, *p* < 0.001; Table 1).

In the whole study cohort, circulating LBP was associated with younger age (r = −0.156, *p* = 0.04), female sex (mean *±* SD of LBP in men: 24.4 ± 22.6 μg/mL vs. women: 30.3 ± 20.2 μg/mL; *p* = 0.01) and greater body adiposity, as evaluated by means of BMI and waist circumference (r = 0.472, *p* < 0.001 and r = 0.40, *p* < 0.001, respectively).

Contrariwise, LBP was lower in T2DM individuals than in non-diabetic individuals (median (25–75° range) LBP: 14.4 (9.1–23.7) μg/mL vs. 24.4 (17.2–44.5) μL/mL, *p* < 0.001). Accordingly, serum LBP levels negatively correlated with FBG (r = −0.19, *p* = 0.01), HbA1c (r = −0.26, *p* = 0.003), diabetes duration (r = −0.20, *p* = 0.034), and albuminuria (r = 0.29, *p* = 0.042) in the bivariate correlation analysis.

The association between lower LBP and the presence of T2DM (β coefficient: −0.061, *p* < 0.001, OR: 0.941, 95% C.I.: 0.915–0.966) was confirmed to be statistically significant after adjustment for potential confounders, such as age, sex, waist circumference, and obesity, in the multivariate logistic regression analysis (Table 2).

In additional analyses considering LBP as a dependent variable, T2DM was shown to independently predict lower LBP levels after adjusting for other covariates, such as sex, age, waist circumference, and obesity (Supplementary Table S1). The stepwise procedure applied to this multivariate linear regression model demonstrated that the diagnosis of T2DM was the major determinant of LBP concentration, which was considered as the dependent variable, regardless of sex, age, waist circumference, and obesity (T2DM unstandardized β: −17.435, standard error: 3.195, standardized β: −5.456, *p*-value < 0.001; R^2^ of the model: 0.168).

After stratifying the study sample according to the presence of obesity, similar results were obtained. Within the obese subgroup (*n* = 121), LBP positively correlated with BMI (r = 0.33, *p* < 0.001) and waist circumference (r = 0.24, *p* = 0.019), but was inversely associated with FBG (r = −0.28, *p* = 0.003) and HbA1c (r = −0.21, *p* = 0.035).

In obese subjects, lower LBP was associated with the presence of T2DM regardless of age and sex in a multivariate linear regression model considering LBP concentration as the dependent variable and the diagnosis of T2DM, age, and sex as covariates (T2DM unstandardized β: −20.017, standard error: 4.944, standardized β: −0.39, *p*-value < 0.001; R^2^ of the model: 0.14).

### 2.2. LBP and NAFLD

Within this study sample, 81 obese individuals underwent surgery intervention for sleeve gastrectomy as a clinical indication, and intra-operative liver biopsy was performed in 47 patients for suspected NAFLD.

**Figure 1:**
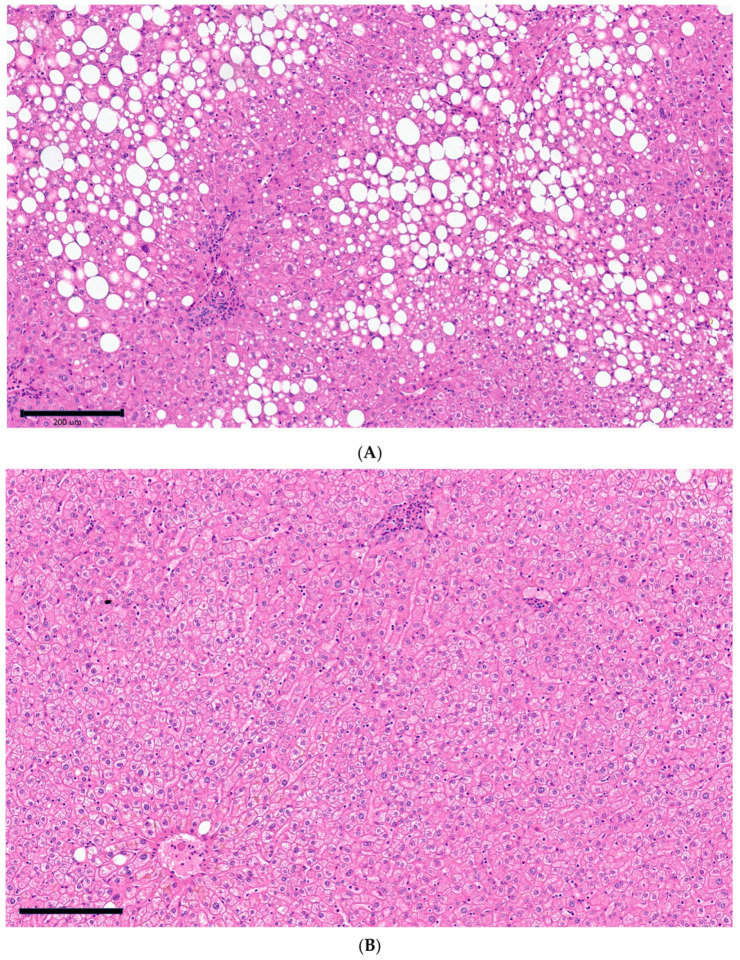
Images showing liver histology of two representative study participants with (**A**) non-alcoholic fatty liver disease (NAFLD) or (**B**) normal liver. Patient (**A**): NAFLD (NAS) activity score: 4, staging: 0; SAF score: steatosis: 3, ballooning: 0, lobular inflammation: 0. Patient (**B**): normal liver tissue. Images viewed under ×200 magnification; hematoxylin and eosin staining.

NAFLD/NASH was diagnosed in 76.7% of cases (36/47; 23.4% no NAFLD, 27.7% mild, 21.3% moderate, and 27.7% severe liver steatosis; Figure 1).

As for intra-hepatocyte lipid accumulation, circulating LBP levels inversely correlated with the percentage of micro-vesicular steatosis (r = −0.38, *p* = 0.024). Low LBP levels were associated with the presence of steatosis (NAS score for steatosis ≥ 1) in the univariate logistic analysis (β = −1.971, OR = 0.139, 95% C.I. = 0.032–0.614, *p* = 0.009), and this relationship remained statistically significant in the multivariate logistic regression model adjusted for potential confounders, such as age, sex, BMI, and diagnosis of T2DM (Table 3).

**Figure 2:**
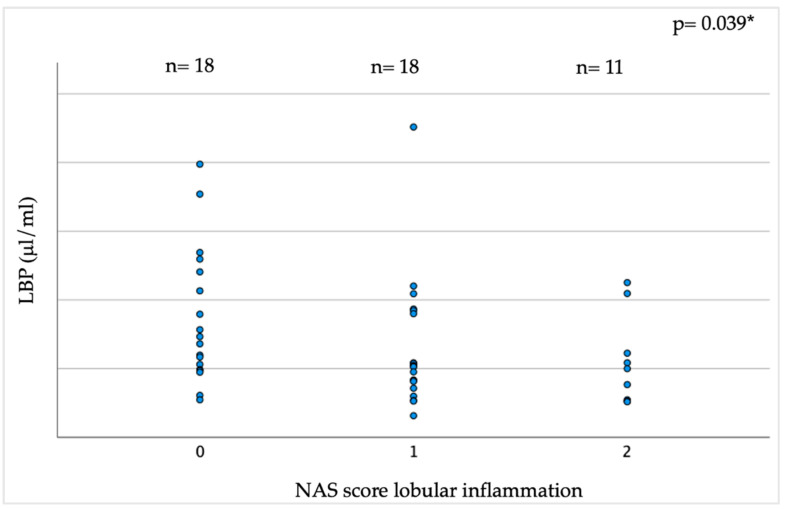
Serum LBP concentration in relation to NAS score for lobular inflammation in liver biopsy. * Spearman’s coefficient. Abbreviations: LBP: lipopolysaccharide-binding protein; NAS: NASH activity score.

Individuals with lobular inflammation, as expressed by an NAS score for lobular inflammation ≥ 1, had lower circulating LBP levels than those without inflammation (24.64 ± 17.1 vs. 34.5 ± 19.58 μL/mL, *p* = 0.035); LBP decreased across NAS inflammation degrees in a dose-dependent manner, as shown in Figure 2 (mean ± SD of LBP in individuals with NAS score 0: 34.5 ± 19.6 μL/mL; NAS score 1: 25.7 ± 18.8 μL/mL; NAS score 2: 22.2 ± 13.1 μL/mL; r = −0.30, *p* = 0.039).

Within the subgroup of patients with circulating LBP concentration below the median value, 77.3% had liver inflammation, whereas this prevalence dropped to 48% among individuals with upper median LBP levels. Thus, having lower LBP was associated with lobular inflammation in the liver biopsy, with an OR of 1.6 (95% C.I. = 1.1–2.6, *p* = 0.03) in the *χ*-squared test.

No significant correlation was found between LBP and liver fibrosis.

### 2.3. LBP and Visceral Adipose Tissue Inflammation

Visceral adipose tissue (VAT) biopsies at the omentum level were obtained in all obese individuals undergoing bariatric surgery (*n* = 81), and immunohistochemistry and gene expression analyses were performed on the VAT fragments to explore the presence of signatures of chronic adipose tissue inflammation.

**Figure 3:**
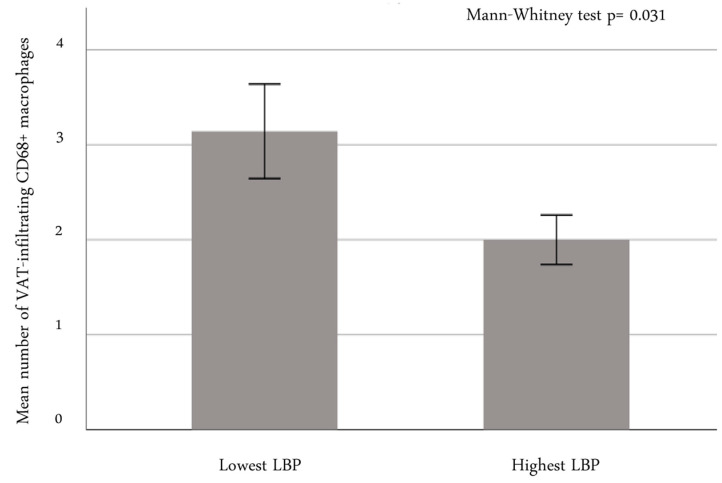
Number of CD68+ cells infiltrating the VAT in relation to serum LBP subgroup. Mean ± standard error. Mann–Whitney test was calculated. Abbreviations: LBP: lipopolysaccharide-binding protein; VAT: visceral adipose tissue.

Circulating LBP levels were inversely associated with signs of chronic inflammation, as identified by the presence and extension of macrophage infiltration, which was expressed as the percentage of CD68+ cells/field (r = −0.21, *p* = 0.049). When dividing the whole study sample into two subgroups according to the median LBP value, we found that patients belonging to the group with the lowest serum LBP levels had a greater number of CD68+ cells infiltrating the VAT than those in the subgroup with the highest LBP levels (*p* = 0.031; Figure 3).

Finally, serum LBP also correlated with the VAT mRNA expression levels of genes associated with hypoxia, i.e., HIF1α (r = −0.29, *p* = 0.008); inflammation, such as TIMP1 (r = −0.28, *p* = 0.013), Netrin1 (r = −0.26, *p* = 0.02), and PARP1 (r = −0.293, *p* = 0.008); and apoptosis, such as Caspase 7 (r = −0.25, *p* = 0.021).

## 3. Discussion

In this study, we demonstrated that reduced circulating LBP levels correlated with the presence of NAFLD and metabolic diseases in an adult obese population. The main finding of this investigation is that, although LBP concentration rises with increasing body adiposity and central fat distribution, in obese individuals, LBP has a negative predictive value for diabetes, histology-proven NAFLD, and VAT inflammation.

In the last decade, the bulk of evidence suggested a pivotal role of impaired gut environment, microbial overgrowth, and endotoxemia in the breakdown of metabolic homeostasis, driven, for the most part, by triggered chronic low-grade inflammation, lipotoxicity, and insulin resistance [23].

In this context, the LPS/LBP axis has emerged as a potential mediator in the progression from chronic positive energy balance and an unbalanced diet composition to clinically overt metabolic disorders and NAFLD [9,10]. When endotoxemia occurs, LPS plays a direct role in triggering liver lipogenesis and enhancing intrahepatic inflammatory responses. Thus, its specific transporter LBP has been assumed for a long time to be a proxy of serum LPS levels, being more stable in the bloodstream and easier to measure than LPS itself, and to concur with LPS-mediated inflammatory damage. This hypothesis was corroborated by data from several studies on increased LBP in obese individuals [15,16,17] and its correlation with atherosclerotic disease [17,24].

However, other studies conducted in either elderly or young populations did not confirm these findings. Circulating levels of LBP did not associate significantly with metabolic abnormalities in studies conducted in children and adolescents with obesity [18] and in adult subjects, whose gut microbiota and short-chain fatty acid characterization was also available [19]. Of note, in longitudinal investigations, LBP was associated with parameters of body adiposity in children but was neither correlated with metabolic alterations nor predicted the development of dysmetabolic disease later in life [20].

Recent evidence showed that higher circulating LBP concentrations were associated with lower cardiovascular risk in older adults [21]. These overall observations are in line with our study findings, which confirmed a tight association between LBP and the obese phenotype but did not reveal any relationship with overt metabolic diseases. Nonetheless, high LBP levels somewhat protected obese individuals from abnormal glucose metabolism and NAFLD, regardless of age, sex, BMI, and classical risk factors.

In line with our results, previous investigations conducted in non-diabetic men showed that circulating LBP predicted impaired fasting glucose development later in life, but this association was largely mediated by changes in truncal fat deposition [25]. Additionally, LBP was not an independent predictor of T2D in a 5-year nested case–control study conducted with over 3500 individuals [26]. A recent meta-analysis of interventional studies found an association between modulation of dietary fiber content and serum LPS but not LBP concentrations [27]; the detection of bacteremia was not even associated with LBP level in T2D patients in other studies [28].

LPS was shown to be a causal factor for hypercoagulability in T2D [29]; thus, LPS-induced thrombotic propensity and amyloid fibril formation were reversed in vitro by adding LBP to the plasma of T2D individuals [29].

Indeed, LPS is largely shown to trigger liver damage associated with obesity through increased gut permeability—the so-called leaky gut—and endotoxemia [23].

In this scenario, LBP and lipoproteins, such as HDL [30], share the capability of binding and delivering LPS into the liver. In particular, LBP, besides activating the CD14/TLR4 downstream pathway, which triggers the inflammatory cascade, may offload LPS onto lipoproteins, facilitating their transportation to the liver for elimination [31]. In a brilliant investigation, Han and collaborators recently demonstrated that the intestine-derived HDL subspecies HDL3 traverses the portal vein in a complex with LBP and prevents LPS from binding and activating liver macrophages, thereby promoting extracellular LPS inactivation [30]. Thus, these alternative pathways, aimed at bacterial toxin clearance, do not provoke a strong inflammatory response, ultimately offering protection against metabolic diseases [30,31].

Indeed, although LPS and LBP parallelly increase in obesity [15,16,17,18,19], LBP, unlike LPS, may exert protective effects against endotoxemia-mediated liver injury and, more generally, obesity-related metabolic complications. All these data may provide mechanistic support to our finding of lower LBP levels in obese patients with NAFLD. In our study, among obese individuals, those with NAFLD had lower LBP levels in the multivariate adjusted regression models; in these patients, the lower the LBP level, the worse the hepatic histological parameters found in the liver biopsy in terms of both steatosis percentage and hepatic inflammatory damage. Further data supporting a potential protective role of LBP in metabolic diseases come from experimental rodent models, where liver LBP gene downregulation resulted in a significant increase in the hepatic expression of markers of inflammatory liver injury, regardless of LPS levels [22].

In our study, we also demonstrated an inverse association between LBP and tissue signatures of VAT inflammation, as detected by both immunohistochemistry and gene expression analyses. VAT-associated metabolic dysfunction and inflammation are leading causes of NAFLD [32] and are also correlated with biomarkers of altered gut homeostasis, such as impaired circulating neurotensin levels, in obese individuals [33,34].

**Figure 4:**
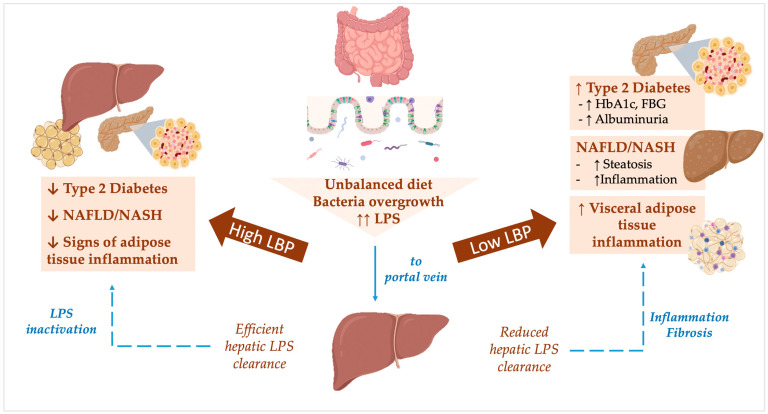
Potential mechanisms associating lower lipopolysaccharide-binding protein (LBP) levels with dysmetabolic conditions in obesity. In obesity, bacterial overgrowth and LPS release into the portal vein trigger systemic inflammation and stimulate pro-inflammatory responses in the liver, resulting in an increased risk of metabolic diseases and NAFLD. LBP may improve hepatic LPS clearance and hide LPS from recognition by TLR4+ macrophages, thus protecting individuals from obesity-associated metabolic disturbances. Dotted arrows show potential metabolic pathways linking LBP levels to metabolic disease in obesity. Abbreviations: NAFLD: non-alcoholic fatty liver disease; NASH: non-alcoholic steatohepatitis; LPS: lipopolysaccharide; LBP: LPS-binding protein; HbA1c: glycosylated hemoglobin; FBG: fasting blood glucose.

A major limitation of this study is the cross-sectional design, which does not allow us to establish a mechanistic nexus underlying our results. However, growing experimental evidence may suggest that, in obesity, chronic endotoxemia stimulates LBP production, which facilitates LPS degradation in the liver. In individuals in whom this adaptive response is preserved (i.e., those with the highest LBP levels), the obese phenotype might not be accompanied by metabolic consequences of chronic inflammation, lipotoxicity, and insulin resistance, and they are less prone to NAFLD and T2D onset. Differently, when a greater LPS concentration fails to stimulate sufficient hepatic response and LBP synthesis/release, LPS clearance may be altered, thus prolonging and exacerbating the inflammatory cascade associated with endotoxemia. The results of this study and the potential mechanisms underlying our findings are summarized in Figure 4.

For this investigation, we compared circulating LBP levels among individuals at high risk of metabolic diseases with or without clinically overt dysmetabolic disorders, who were all referred to our Diabetes and Endocrinology outpatient clinic. Thus, a study weakness may be the lack of a proper control group of normal-weight healthy individuals recruited from the general population. In our study sample, the prevalence of female participants was higher in the obese group than the non-obese group. Since it could introduce a potential bias, all study analyses were adjusted for sex, showing that the main study findings were sex independent. Similarly, sex was not associated with any study outcome in the multivariate regression models.

This study has several strengths. First, it was conducted in an extremely well-characterized population of individuals with or without obesity who were at high risk of metabolic diseases. A relevant proportion of the study participants underwent VAT and coupled VAT-liver biopsy, providing histological data on metabolic/inflammatory features in organs that are central to metabolic regulation. Finally, to the best of our knowledge, this is the first study investigating the relationships of LBP with T2D, histologically proven VAT, and liver metabolic diseases in human obesity.

In conclusion, our study findings add insights into metabolic disorders associated with obesity, with a focus on alterations in the gut–liver–adipose tissue system, in which the LPS/LBP axis may be implicated with unexpected roles, thus opening new scenarios for risk stratification strategies and therapeutic approaches.

## 4. Materials and Methods

### 4.1. Study Population and Clinical Evaluations

For this cross-sectional investigation, we recruited 186 individuals (M/F: 81/105; mean **±** SD age: 47 ± 10.4 years; BMI: 35.5 ± 8.6 kg/m^2^) who were referred to the Diabetes and Endocrinology outpatient clinic at Sapienza University, Rome, Italy, for metabolic evaluations. To participate in this study, individuals had to meet the following inclusion/exclusion criteria: male or female sex; age between 20 and 65 years; no history of excessive alcohol consumption, defined as daily alcohol intake exceeding 30 g in men and 20 g in women; negative results for hepatitis B surface antigen and hepatitis C virus antibody; no prior history of cirrhosis or other liver diseases (such as hemochromatosis, autoimmune hepatitis, and Wilson’s disease); and no ongoing treatment with medications known to induce liver steatosis (e.g., corticosteroids, estrogens, methotrexate, tetracycline, calcium channel blockers, or amiodarone).

Study participants underwent standard medical history collection and clinical evaluations. Individuals without overt T2DM diagnosis underwent standard oral glucose tolerance test (OGTT) [35]. Anthropometric measurements included recording weight and height, with lightweight clothing and barefoot, for body mass index calculation (BMI, kg/m^2^). Waist circumference (in centimeters) was measured between the 12th rib and the iliac crest. Systemic systolic (SBP) and diastolic (DBP) blood pressure readings were obtained following a 5 min rest period, with three measurements taken, and the average of the second and third measurements was utilized in the analyses.

Individuals with obesity and clinical indication for bariatric surgery underwent sleeve gastrectomy in accordance with the American Association for the Study of Liver Diseases (AASLD) recommendations [36] and VAT–liver biopsies were obtained intra-procedurally.

### 4.2. Laboratory Procedures

Venous blood samples were collected after a 12 h fast for both clinical and experimental assessments. Fasting blood glucose (FBG, mg/dL), glycosylated hemoglobin (%–mmol/mol), total cholesterol (mg/dL), high-density lipoprotein cholesterol (HDL, mg/dL), triglycerides (mg/dL), aspartate aminotransferase (AST, IU/L), alanine aminotransferase (ALT, IU/L), gamma-glutamyl transpeptidase (GGT, mg/dL), and C-reactive protein (ng/mL) were measured using standard methods at Sapienza University. Low-density lipoprotein (LDL) cholesterol value was obtained using the Friedewald formula. Serum LBP levels were determined using a human enzyme-linked immunosorbent assay (ELISA) kit (HK315-02, HyCult Biotech Inc., Uden, The Netherlands) according to the manufacturer’s specifications. Briefly, the samples were diluted according to the instructions and LBP was measured at 450 nm using a spectrophotometer. The detection limit for LBP was 4.4 μg/mL.

### 4.3. Histology and Gene Expression Analyses

Liver and adipose tissue histological evaluations were conducted on hepatic and VAT biopsies obtained from individuals undergoing laparoscopic sleeve gastrectomy, according to the AASLD standards [36]. The liver specimens were fixed in buffered formalin for 2 to 4 h, embedded in paraffin, and exposed to hematoxylin and eosin staining, along with Masson’s trichrome stains; all histological assessments were carried out by an experienced clinical pathologist blinded to the patients’ medical history and biochemistry. The biopsy samples were required to reach a minimum length of 15 mm or show the presence of 10 complete portal tracts. The liver biopsies were classified according to the presence of non-alcoholic steatohepatitis (NASH) using Brunt’s definition and graded based on the NAFLD activity score (NAS) [37]. Fibrosis was quantified using the NASH Clinical Research Network Scoring System Definition.

For VAT histology and gene expression analyses, biopsies were collected from the omentum, fixed with 10% buffered formalin for 24 h, and embedded in paraffin, as details in previous works [32,33]. Hematoxylin and eosin staining, as well as Masson’s trichrome staining, was performed. Collagen fibers were quantified using fast green FCF/Sirius staining. Immunohistochemical staining for CD68 monoclonal antibody (clone M0876, 1:100; Dako, Carpinteria, CA, USA) and CD34 (clone QBEnd/10; Leyca Biosystem, Newcastle, UK) was used to quantify macrophages and micro-vessel density. The results were expressed on a semi-quantitative scale.

Gene expression analysis was performed using total RNA extracted from the FFPE samples, and real-time quantitative PCR was conducted for specific genes. Specifically, we extracted total RNA from the formalin-fixed, paraffin-embedded (FFPE) samples using the RecoverAllTM Total Nucleic Acid Isolation Kit for FFPE (ThermoFisher Scientific, Waltham, MA, USA), as previously described [33].

Then, we detected the PCR products of human genes involved in inflammation, apoptosis, and hypoxia, such as UNC5B, NTN1, IL8, CAV1, MIP1A, MIP2, TIMP1, CASP3, CASP7, HIF-1α, and WISP1, using gene-specific primers and probes labeled with the reporter dye FAM. The criteria for gene selection are explained in detail in our previous investigation [38]. To maintain consistency, GAPDH was used as an internal standard, with a predicted amplicon of 171 bp. The TaqMan real-time quantitative PCR was carried out using an ABI PRISM 7500 Fast Real-Time PCR System (Applied Biosystem in Foster City, CA, USA). The PCR reactions were conducted in triplicate using 96-well plates, with 10 L of ×1 TaqMan Master Mix per well, and the results were assessed using the ABI PRISM 7500 software (Applied Biosystem, Foster City, CA, USA).

### 4.4. Statistics

All statistical analyses were performed using SPSS software version 27.0. Continuous variables are reported as mean ± standard deviation (SD), and categorical variables are reported as percentages in the manuscript and tables. Student’s *t*-test for continuous normally distributed parameters, Mann–Whitney test for non-normally distributed parameters, or χ^2^ test for categorical variables was used to compare the mean values between two independent groups, as appropriate; skewed variables underwent natural logarithmic transformations before inferential analyses were performed. Correlations between the parameters were explored using Pearson’s or Spearman’s coefficient, as appropriate. Multivariate logistic regression analyses were performed to detect independent predictors of T2DM or NAFLD, with the covariates that were significantly associated with the dependent variable in the bivariate correlation analyses being entered into the analyses. Two-sided *p* < 0.05 was considered statistically significant, with a confidence interval of 95%.

## Figures and Tables

**Table 1:** Characteristics of the study sample according to the presence of obesity.

Clinical Parameters	Obese Subjects (*n* = 121)	Non-Obese Subjects (*n* = 65)	*p*-Value
Age (years)	46.5 ± 10.3	48 ± 10.5	0.35
Sex (female *n*, %)	79, 65.3%	26, 40%	<0.001
BMI (Kg/m^2^)	40.7 ± 6.2	26.2 ± 2.5	<0.001
Waist circumference (cm)	122.4 ± 14.8	92.1 ± 11.8	<0.001
SBP (mmHg)	130.1 ± 15.3	123.6 ± 14.5	0.01
DBP (mmHg)	82.8 ± 9.1	78.7 ± 8	0.004
FBG (mg/dL)	108.3 ± 31.4	113.8 ± 35.7	0.32
HbA1c (%, mmol/mol)	5.9 ± 0.99, 41 ± 13	6.30 ± 0.6, 45 ± 17	0.01
Total cholesterol (mg/dL)	191.3 ± 34.3	184.7 ± 36.3	0.25
HDL-c (mg/dL)	47.6 ± 12	52.3 ± 14.3	0.03
LDL-c (mg/dL)	113.7 ± 33.3	106.9 ± 32	0.20
Triglycerides (mg/dL)	142.2 ± 66.2	124.9 ± 71.2	0.11
AST (IU/L)	25.9 ± 12.8	21.8 ± 10.7	0.03
ALT (IU/L)	34.6 ± 23.4	27 ± 20.5	0.03
GGT (IU/L)	32.1 ± 36	28.9 ± 26.3	0.54
CRP (mg/dL)	4.7 ± 4.6	2 ± 3.1	0.01
LBP (μg/mL)	33.4 ± 23.2	16.6 ± 10.7	<0.001
Prevalence of T2DM (%)	30.8%	53.2%	0.003
Prevalence of MS (%)	80%	40%	<0.001

Data are shown as mean values ± standard deviation (SD) or percentages. Abbreviations: BMI: body mass index; SBP: systolic blood pressure; DBP: diastolic blood pressure; FBG: fasting blood glucose; HbA1c: glycosylated hemoglobin; HDL-C: high-density lipoprotein; LDL: low-density lipoprotein; AST: aspartate aminotransferase; ALT: alanine aminotransferase; GGT: gamma-glutamyl transferase; CRP: C-reactive protein; LBP: lipopolysaccharide-binding protein; T2DM: type 2 diabetes mellitus; MS: metabolic syndrome.

**Table 2:** Multivariate logistic regression analysis for presence of T2DM.

	β Coefficient	*p*-Value	Odds Ratio	95% C.I.
LBP (μg/mL)	−0.058	<0.001	0.943	0.913–0.974
Age (years)	0.069	0.002	1.072	1.026–1.120
Sex (male vs. female)	−1.541	<0.001	0.214	0.089–0.517
Waist circumference (cm)	−0.020	0.219	0.980	0.950–1.012
Obesity (yes vs. no)	0.500	0.440	1.648	0.464–5.854

The presence of T2DM is the dependent variable. Cox and Snell R^2^ of the model: 0.322. Abbreviation: LBP: lipopolysaccharide-binding protein.

**Table 3:** Multivariate logistic regression analysis for NAFLD.

	β Coefficient	*p*-Value	Odds Ratio	95% C.I.
LBP (μg/mL)	−1.87	0.047	0.155	0.025–0.977
Age (years)	−0.042	0.497	0.958	0.848–1.083
Sex (male vs. female)	1.390	0.096	10.121	0.669–154.46
BMI (kg/m^2^)	0.211	0.157	1.235	0.922–1.655
T2DM diagnosis (yes vs. no)	−22.27	0.999	0.000	0.000

The presence of hepatosteatosis (yes vs. no) is the dependent variable. Cox and Snell R^2^ of the model: 0.415. Abbreviations: LBP: lipopolysaccharide-binding protein; BMI: body mass index; T2DM: type 2 diabetes mellitus.

## Data Availability

The data presented in this study are available from the corresponding author upon request. The data are not publicly available due to privacy restrictions and lack of specific patient consent.

## Supplementary Materials

The supporting information can be downloaded at https://www.mdpi.com/article/10.3390/ijms242417174/s1.
