# Supplementary material for: Reduced Lipopolysaccharide-Binding Protein (LBP) Levels Are Associated with Non-Alcoholic Fatty Liver Disease (NAFLD) and Adipose Inflammation in Human Obesity
Source: Int J Mol Sci. 2023 Dec 6;24(24):17174. doi: 10.3390/ijms242417174 (PMC10742626; doi:10.3390/ijms242417174)
Supplement: Supplementary file 1 [file ijms-24-17174-s001.zip › ijms-2721230-supplementary.pdf]

Supplementary Table S1. Multivariable linear regression analysis.

	Unstandardized β coefficient	Standard Error	Standardized β coefficient	P value	95% C.I.
Age (years)	0.113	0.154	0.054	0.465	-0.192 - 0.418
Sex (male vs. female)	-4.13	3.376	-0.098	0.223	-10.804 -2.543
Waist circumference (cm)	-0.090	0.114	0.085	0.432	-0.135- 0.314
Obesity (yes vs. no)	12.136	4.814	0.277	0.013	2.620 - 21.652
T2DM (yes vs. no)	-15.449	3.418	--0.364	<0.001	-22.206 - -8.692

Circulating LBP concentration is the dependent variable.

Cox and Snell R^2 of the model: 0.280.

Abbreviations: LBP: lipopolysaccharides binding protein; T2DM: type 2 diabetes mellitus.
